# The Fourth Dimension of Biochemical Pathways

**DOI:** 10.1371/journal.pbio.0060151

**Published:** 2008-06-17

**Authors:** Richard Robinson

Even on a plain wall chart, the intricacies of a cell's biochemical pathways can boggle the mind. Hundreds of interweaving routes create and consume thousands of intermediate compounds, which are regulated by a dizzying number of enzymes at every step—a drop in nutrient A turns on pathway B to make intermediate C that is converted to regulator D that stimulates gene E that creates enzyme F to divert intermediate G into pathway H to…whew!…make nutrient A. London's famously complex subway system is a piker compared to even the simplest cell.

But a static map can't depict the complexity of a subway system in motion, and a wall chart can't capture the four-dimensional dynamism of a cell in action, because neither one captures the crucial dimension of time. It matters not only where a train is going, but when it will get there, and it matters not only whether a pathway can produce a nutrient, but how quickly it responds when the nutrient is depleted. In a new study, Chen-Shan Chin, Victor Chubukov, Hao Li, and colleagues begin to address this problem by using a novel method to track the time course of a cell's response to depletion of the amino acid leucine. They show that the time responses of upstream and downstream segments differ dramatically, and they go on to develop a mathematical model that predicts the response of the pathway to experimental perturbations.

In yeast, the raw material for leucine synthesis is pyruvate, which is formed during breakdown of sugar. Like factory floor workers, a row of nine enzymes add and remove atoms in succession, each passing its product onto the next for further modification. To study the dynamics of this pathway in vivo and in real time, the authors used multiple strains of yeast, each of which had one enzyme's gene fluorescently tagged, so that as that gene was expressed, they could identify when and how much of each enzyme was made.

The authors grew their cells in a set of culture vessels with computer-controlled pumps linked to a fluorescently activated cell sorting device, allowing them to determine not just how cells respond on average to leucine depletion, but how individual cells differ in the speed and degree of enzyme expression. Since their yeast were dividing throughout the experiment, and since mature and growing cells differ significantly in their metabolic responses, they added a second tag (based on cell wall differences) that allowed them to separate mother and daughter cells.

**Figure:**
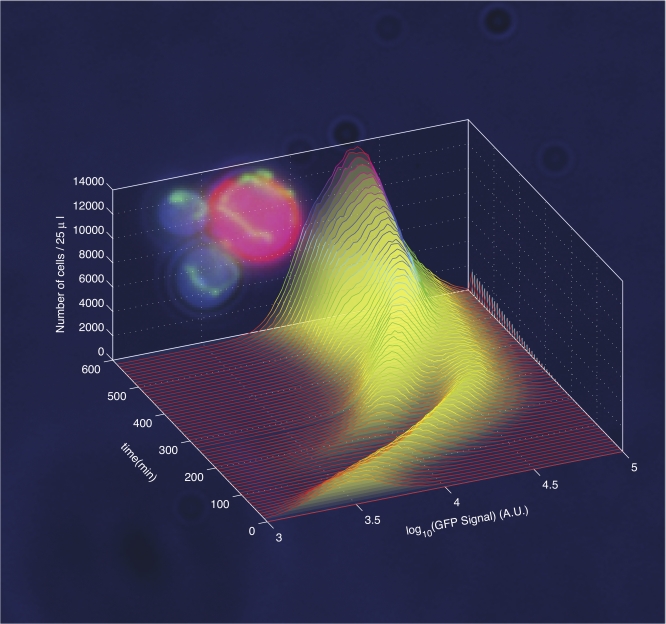
Dynamics of gene induction represented by a three-dimensional histogram. In response to leucine depletion, yeast cells produce GFP-tagged Leu1 protein, and the distribution of fluorescence shifts to the right. The shape of the distribution also changes as the population divides. The background is a microscopy image of these cells during cell division.

One of the intermediate metabolites in leucine synthesis, α-isopropylmalate (αIPM), plays a crucial role in regulating the activity of the pathway. When αIPM binds to the transcription factor leu3, leu3 turns on the genes for the pathway's enzymes. Thus, as αIPM builds up, more enzymes are churned out to process it into leucine. However, the enzyme that makes αIPM is inhibited by leucine; thus, when leucine is abundant in the environment, little αIPM is made, and little leucine is synthesized by the yeast.

The authors found that the temporal response to leucine depletion was quite different for enzymes before and after the αIPM checkpoint. The genes for those early in the path were turned on quickly upon leucine deprivation, but expression increased rather weakly, by only 2- to 5-fold, and production had reached a steady state within an hour. In contrast, the expression rate of genes for two of the three downstream enzymes climbed steeply only after 1 hour, and after 6 hours, expression had increased by a factor of 20.

The authors showed that the induction of the upstream genes depended less on the transcription factor leu3 and more on other, more general, responses to amino acid starvation, hence their rapid but low-level response to depletion of this single amino acid. The two downstream genes, however, were almost entirely controlled by leu3—depletion of leucine released the αIPM-producing enzyme to make more αIPM, which activated leu3 to strongly induce the two genes.

They then constructed a mathematical model that matched the initial data, and used it to predict the system's temporal response to excess αIPM or overexpression of one of the downstream enzymes. The model accurately predicted the time course and amplitude of the cell culture's response in each case.

The authors argue that the strong control by αIPM over the downstream enzymes provides the cell with two advantages. By keeping production very low in the presence of leucine, the cell avoids wasting resources when leucine is abundant. By boosting production dramatically when leucine is scarce, the cell minimizes the delay in growth that would otherwise occur. They note that several other pathways share this same key control feature, suggesting it may be a general design feature to optimize the dynamic response to nutrient scarcity.

